# Tunneling-Magnetoresistance Ratio Comparison of MgO-Based Perpendicular-Magnetic-Tunneling-Junction Spin Valve Between Top and Bottom Co_2_Fe_6_B_2_ Free Layer Structure

**DOI:** 10.1186/s11671-016-1637-9

**Published:** 2016-09-27

**Authors:** Du-Yeong Lee, Seung-Eun Lee, Tae-Hun Shim, Jea-Gun Park

**Affiliations:** Department of Electronics and Computer Engineering, Hanyang University, Seoul, 04763 Republic of Korea

**Keywords:** p-MTJ, BEOL, TMR ratio, Pt diffusion, Top and bottom free layer

## Abstract

For the perpendicular-magnetic-tunneling-junction (p-MTJ) spin valve with a nanoscale-thick bottom Co_2_Fe_6_B_2_ free layer ex situ annealed at 400 °C, which has been used as a common p-MTJ structure, the Pt atoms of the Pt buffer layer diffused into the MgO tunneling barrier. This transformed the MgO tunneling barrier from a body-centered cubic (b.c.c) crystallized layer into a mixture of b.c.c, face-centered cubic, and amorphous layers and rapidly decreased the tunneling-magnetoresistance (TMR) ratio. The p-MTJ spin valve with a nanoscale-thick top Co_2_Fe_6_B_2_ free layer could prevent the Pt atoms diffusing into the MgO tunneling barrier during ex situ annealing at 400 °C because of non-necessity of a Pt buffer layer, demonstrating the TMR ratio of ~143 %.

**Electronic supplementary material:**

The online version of this article (doi:10.1186/s11671-016-1637-9) contains supplementary material, which is available to authorized users.

## Background

Perpendicular spin-transfer-torque magnetic random access memory (p-STT MRAM) has recently been greatly researched because it would overcome the scaling-down limitation of less than 10 nm of current dynamic random access memory (DRAM) [1, 2]. To achieve a tera-bit integration memory cell, three critical device performance parameters of CoFeB/MgO-based perpendicular-magnetic-tunneling-junction (p-MTJ) spin valves in a p-STT MRAM cell have been rapidly improved: tunneling-magnetoresistance (TMR) ratio, thermal stability (*Δ*), and switching current density (*J*_c_) [3–6]. In particular, the structure design of a p-MTJ spin valve (i.e., a p-MTJ spin valve with a nanoscale-thick top free layer or a p-MTJ spin valve with a nanoscale-thick bottom free layer) has intensively been studied to achieve a higher TMR ratio at the back end of line (BEOL) temperature of 400 °C [7–10]. Note that the BEOL temperature of 400 °C is required to integrate memory cells [11]. In addition, for a p-MTJ spin valve with a nanoscale-thick top free layer, the CoFeB free layer is located above the synthetic antiferromagnetic (SyAF) layer in a p-MTJ spin valve, while for a p-MTJ spin valve with a nanoscale-thick bottom free layer, the CoFeB free layer is located below the SyAF layer in a p-MTJ spin valve. In our study, the dependencies of the TMR ratio at the BEOL temperatures of 350 and 400 °C on the structure of a p-MTJ spin valve were investigated. In addition, the reason the structure difference for a p-MTJ spin valve directly influences the TMR ratio was characterized by static magnetization behavior, crystallinity, and the depth profile of atomic composition of p-MTJ spin valves.

## Methods

**Fig. 1:**
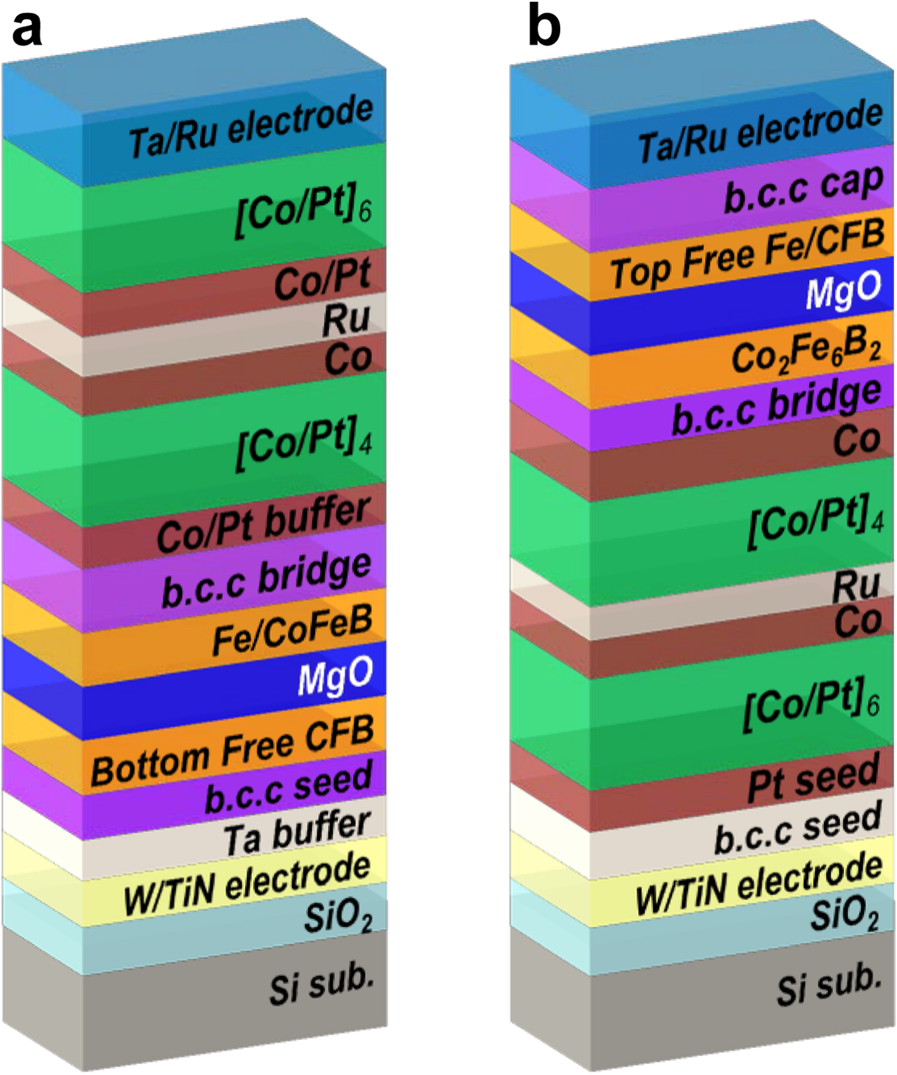
p-MTJ spin valve structure. **a** p-MTJ spin valve with a nanoscale-thick bottom Co_2_Fe_6_B_2_ free layer. **b** p-MTJ spin valve with a nanoscale-thick top Co_2_Fe_6_B_2_ free layer

CoFeB/MgO-based p-MTJ spin valves with a [Co/Pt]_n_-based SyAF layer were prepared using a 12-in. multi-chamber sputtering system under a high vacuum of less than 1 × 10^−8^ torr without breaking vacuum. Two types of p-MTJ spin valves were fabricated on 12-in. SiO_2_ wafers, as shown in Fig. 1: One type was a p-MTJ spin valve with a bottom Co_2_Fe_6_B_2_ free layer and the other type was a p-MTJ spin valve with a top Co_2_Fe_6_B_2_ free layer. As shown in Fig. 1a, p-MTJ spin valves with a bottom Co_2_Fe_6_B_2_ free layer were deposited with vertically stacked layers from the SiO_2_ substrate, W/TiN bottom electrode/Ta buffer layer/body-centered-cubic (b.c.c) seed layer/Co_2_Fe_6_B_2_ (1.0 nm) free layer/MgO (1.15 nm) tunnel barrier/Fe (0.4 nm)/Co_2_Fe_6_B_2_ (1.0 nm) pinned layer/b.c.c bridge layer/Co/Pt (2.0 nm) buffer layer/lower [Co/Pt]_4_-SyAF layer/Co/Ru spacer/Co/Pt/upper [Co/Pt]_6_-SyAF layer/Ta/Ru top electrode. On the other hand, as shown in Fig. 1b, p-MTJ spin valves with a top Co_2_Fe_6_B_2_ free layer were deposited with vertically stacked layers from the SiO_2_ substrate, W/TiN bottom electrode/Ta buffer layer/Pt (3.0 nm) seed layer/upper [Co/Pt]_6_-SyAF layer/Co/Ru spacer/Co/Pt/lower [Co/Pt]_4_-SyAF layer/Co/b.c.c bridge layer/Co_2_Fe_6_B_2_ (1.0 nm) pinned layer/MgO (1.15 nm) tunnel barrier/Fe (0.4 nm)/Co_2_Fe_6_B_2_ (1.0 nm) free layer/b.c.c capping layer/Ta/Ru top electrode. The b.c.c metal has a lattice constant of 3.16 Å [3]. In addition, a 0.4-nm-thick Fe layer was inserted between the MgO tunneling barrier and the Co_2_Fe_6_B_2_ layer to obtain a higher TMR ratio [12]. All p-MTJ spin valves were subject to ex situ annealing at 350 or 400 °C under a perpendicular magnetic field of 3 T.

## Results and Discussion

**Fig. 2:**
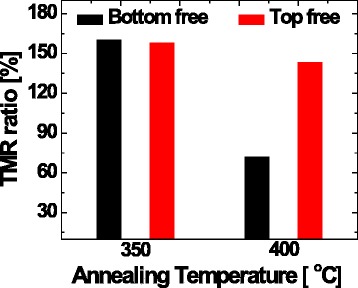
TMR ratio depending on the p-MTJ spin valve structure and ex situ annealing temperature. (*Black*) p-MTJ spin valve with a nanoscale-thick bottom Co_2_Fe_6_B_2_ free layer and (*red*) p-MTJ spin valve with a nanoscale-thick top Co_2_Fe_6_B_2_ free layer ex situ annealed at 350 and 400 °C

The TMR ratio for p-MTJ spin valves depending on the p-MTJ spin valve structure was estimated by using current-in-plane tunneling (CIPT) at room temperature [13], as shown in Fig. 2. At an ex situ annealing temperature of 350 °C, the TMR ratio was ~160 % for the p-MTJ spin valve with a nanoscale-thick bottom Co_2_Fe_6_B_2_ free layer and ~158 % for the p-MTJ spin valve with a nanoscale-thick top Co_2_Fe_6_B_2_ free layer, indicating the TMR ratio did not depend on the p-MTJ spin valve structure. However, the TMR ratio for the p-MTJ spin valve with a nanoscale-thick bottom Co_2_Fe_6_B_2_ free layer rapidly decreased from ~160 to ~72 % when the ex situ annealing temperature increased from 350 to 400 °C. Otherwise, the TMR ratio for the p-MTJ spin valve with a nanoscale-thick top Co_2_Fe_6_B_2_ free layer slightly decreased from ~158 to ~143 % when the ex situ annealing temperature increased from 350 to 400 °C. These results imply that the TMR ratio for the p-MTJ spin valve with a nanoscale-thick bottom Co_2_Fe_6_B_2_ free layer (~88 %) decreased by approximately six more than that for the p-MTJ spin valve with a nanoscale-thick top Co_2_Fe_6_B_2_ free layer (~15 %).

**Fig. 3:**
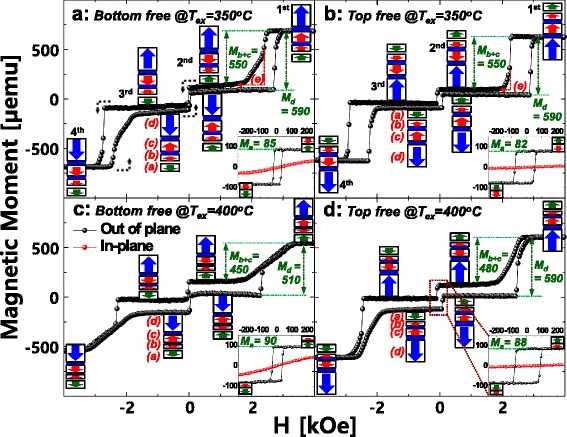
Dependency of *M-H* curves on the p-MTJ spin valve structure and ex situ annealing temperature. *M-H* curves in figures describe the static perpendicular magnetization behaviors of Co_2_Fe_6_B_2_ free layer (**a**), Co_2_Fe_6_B_2_ pinned layer (**b**), lower SyAF layer (**c**), and upper SyAF layer (**d**). *M-H* curves in the inlets of figures only correspond to the static i-PMA behavior of Co_2_Fe_6_B_2_ free layer, where the *black M-H* curves represent i-PMA behavior and the *red M-H* curves represent the in-plane behavior. **a** p-MTJ spin valve with a nanoscale-thick bottom Co_2_Fe_6_B_2_ free layer ex situ annealed at 350 °C, **b** p-MTJ spin valve with a nanoscale-thick top Co_2_Fe_6_B_2_ free layer ex situ annealed at 350 °C, **c** p-MTJ spin valve with a nanoscale-thick bottom Co_2_Fe_6_B_2_ free layer ex situ annealed at 400 °C, and **d** p-MTJ spin valve with a nanoscale-thick top Co_2_Fe_6_B_2_ free layer ex situ annealed at 400 °C

First, to reveal the reason the TMR ratio at an ex situ annealing temperature of 400 °C depends on the p-MTJ spin valve structure, the dependency of the static magnetization behavior of p-MTJ spin valves on the p-MTJ spin valve structure was investigated via the magnetic moment vs. magnetic field (*M-H*) curves measured by a vibrating sampling magnetometer (VSM) at room temperature, as shown in Fig. 3. Both p-MTJ spin valves with nanoscale-thick bottom and top Co_2_Fe_6_B_2_ free layers consisted of four magnetic layers as shown in Fig. 3a, b: Co_2_Fe_6_B_2_ free layer (a), Co_2_Fe_6_B_2_ pinned layer (b), lower SyAF layer (c), and upper SyAF layer (d). In Fig. 3, the length of boxes and vectors corresponds to the magnitude of magnetic moment and the spin-electron direction for a magnetic layer, respectively. Initially, the Co_2_Fe_6_B_2_ pinned layer (b) was ferro-coupled with the lower SyAF layer (c) while the lower SyAF layer (c) was antiferro-coupled with the upper SyAF layer (d) [14, 15]. Thus, when the applied magnetic field is scanned from 0, 300, 0, −300, and 0 Oe, only the spin-electron direction of the Co_2_Fe_6_B_2_ free layer (a) can be rotated along the applied magnetic field direction, as shown in the inlets in Fig. 3. However, when the applied magnetic field is scanned from 0, 3, 0, −3, and 0 kOe, the spin-electron directions of all four magnetic layers can be rotated. For the p-MTJ spin valve with a nanoscale-thick bottom Co_2_Fe_6_B_2_ free layer ex situ annealed at 350 °C, the magnetic moments of the Co_2_Fe_6_B_2_ free layer (a), Co_2_Fe_6_B_2_ pinned layer ferro-coupled with lower SyAF layer (b + c), and upper SyAF layer (d) were 85, 550, and 590 μemu, respectively, as shown in Fig. 3a. In addition, the nanoscale-thick bottom Co_2_Fe_6_B_2_ free layer showed an excellent interface-perpendicular-magnetic-anisotropy (i-PMA) characteristic [16, 17], as shown in the *M-H* curve of the bottom Co_2_Fe_6_B_2_ free layer in the inlet in Fig. 3a. On the other hand, for the p-MTJ spin valve with a nanoscale-thick top Co_2_Fe_6_B_2_ free layer ex situ annealed at 350 °C, the magnetic moments of the Co_2_Fe_6_B_2_ free layer (a), Co_2_Fe_6_B_2_ pinned layer ferro-coupled with lower SyAF layer (b + c), and upper SyAF layer (d) were 82, 550, and 590 μemu, respectively, as shown in Fig. 3b. In addition, the top Co_2_Fe_6_B_2_ free layer showed an excellent i-PMA characteristic, as shown in the *M-H* curve of the top Co_2_Fe_6_B_2_ free layer in the inlet in Fig. 3b. The magnetic moments for the p-MTJ spin valves with a nanoscale-thick bottom Co_2_Fe_6_B_2_ free layer and a top Co_2_Fe_6_B_2_free layer were almost the same, indicating that these p-MTJ spin valves did not significantly differ in terms of static magnetic behavior except a slight degradation in the ferro-coupling strength between the Co_2_Fe_6_B_2_ pinned layer ferro-coupled with the lower SyAF layer (compare (e) in Fig. 3a with (e) in Fig. 3b). However, for the p-MTJ spin valve with a nanoscale-thick bottom Co_2_Fe_6_B_2_ free layer, when the ex situ annealing temperature increased from 350 to 400 °C, the magnetic moment of the bottom Co_2_Fe_6_B_2_ free layer (see the inlets of Fig. 3a, c) slightly increased from 85 to 90 μemu while that of the Co_2_Fe_6_B_2_ pinned layer ferro-coupled with lower SyAF layer (see (b + c) of Fig. 3a, c) decreased from 550 to 450 μemu and that of the upper SyAF layer (see (d) of Fig. 3a, c) decreased from 590 to 510 μemu. These results indicate that i-PMA characteristic (PMA magnetic moment) of the Co_2_Fe_6_B_2_ pinned layer for the p-MTJ spin valve with a nanoscale-thick bottom Co_2_Fe_6_B_2_ free layer was remarkably degraded when the ex situ annealing temperature increased from 350 to 400 °C. Furthermore, for the p-MTJ spin valve with a nanoscale-thick top Co_2_Fe_6_B_2_ free layer, when the ex situ annealing temperature increased from 350 to 400 °C, the magnetic moments increased slightly from 82 to 88 μemu for the top Co_2_Fe_6_B_2_ free layer (see the inlets of Fig. 3b, d) but decreased from 550 to 480 μemu for the Co_2_Fe_6_B_2_ pinned layer ferro-coupled with the lower SyAF layer (see (b + c) of Fig. 3b, d) and at stayed at 590 μemu for the upper SyAF layer (see (d) of Fig. 3c, d). These results imply that i-PMA characteristic (i.e., PMA magnetic moment) slightly degraded for the Co_2_Fe_6_B_2_ pinned layer for the p-MTJ spin valve with a nanoscale-thick top Co_2_Fe_6_B_2_ free layer but barely changed for the SyAF layer. In summary, when the ex situ annealing temperature increased from 350 to 400 °C, i-PMA characteristic degradation (i.e., PMA magnetic moment decrease) of the Co_2_Fe_6_B_2_ pinned layer was much severer for the p-MTJ spin valve with a nanoscale-thick bottom Co_2_Fe_6_B_2_ free layer than the p-MTJ spin valve with a nanoscale-thick top Co_2_Fe_6_B_2_ free layer.

**Fig. 4:**
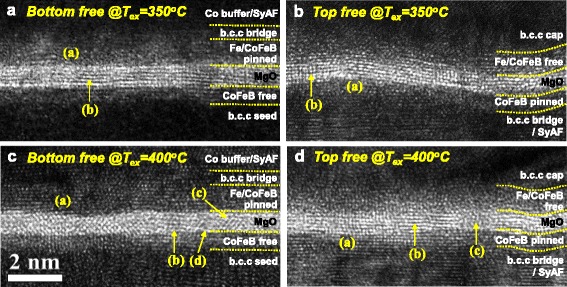
Dependency of MgO tunneling crystallinity on the p-MTJ spin valve structure and ex situ annealing temperature. **a** p-MTJ spin valve with a nanoscale-thick bottom Co_2_Fe_6_B_2_ free layer ex situ annealed at 350 °C, **b** p-MTJ spin valve with a nanoscale-thick top Co_2_Fe_6_B_2_ free layer ex situ annealed at 350 °C, **c** p-MTJ spin valve with a nanoscale-thick bottom Co_2_Fe_6_B_2_ free layer ex situ annealed at 400 °C, and **d** p-MTJ spin valve with a nanoscale-thick top Co_2_Fe_6_B_2_ free layer ex situ annealed at 400 °C

Since the TMR ratio for the p-MTJ spin valves is mainly determined by the i-PMA characteristic (i.e., PMA magnetic moment) of the Co_2_Fe_6_B_2_ pinned layer as well as the b.c.c crystallinity of the MgO tunneling barrier, when the ex situ annealing temperature increased from 350 to 400 °C, the dependency of the MgO tunneling barrier b.c.c crystallinity on the p-MTJ structure (i.e., the p-MTJ with a nanoscale-thick bottom or top Co_2_Fe_6_B_2_ free layer) was investigated as a function of ex situ annealing temperature via cross-sectional transmission electron microscope (x-TEM) observation, as shown in Fig. 4. For the p-MTJ spin valve with a nanoscale-thick bottom Co_2_Fe_6_B_2_ free layer ex situ annealed at 350 °C, the Co_2_Fe_6_B_2_ pinned layer and the MgO tunneling barrier were revealed as an amorphous layer ((a) in Fig. 4a) and a (100) b.c.c crystallized layer ((b) in Fig. 4a) [18–20], respectively. In particular, the MgO tunneling barrier was flatly grown, and the thickness of the MgO tunneling barrier was very uniform. In addition, for the p-MTJ spin valve with a nanoscale-thick top Co_2_Fe_6_B_2_ free layer ex situ annealed at 350 °C, the Co_2_Fe_6_B_2_ pinned layer and the MgO tunneling barrier were also exhibited as an amorphous layer ((a) in Fig. 4b) and (100) b.c.c crystallized layer ((b) in Fig. 4b), respectively. Surprisingly, the MgO tunneling barrier was flexuously grown although the thickness of the MgO tunneling barrier was almost uniform. Note that a flexuous growth of the MgO tunneling barrier in Fig. 4b originated from a thick SyAF layer and could degrade the b.c.c crystallinity of the MgO tunneling barrier. Otherwise, for the p-MTJ spin valve with a nanoscale-thick bottom Co_2_Fe_6_B_2_ free layer, when the ex situ annealing temperature increased from 350 to 400 °C, the Co_2_Fe_6_B_2_ pinned layer was revealed as a (100) b.c.c crystallized layer because of the texturing of the b.c.c bridging layer during ex situ annealing at 400 °C ((a) in Fig. 4c) rather than an amorphous layer, and the MgO tunneling barrier was exposed as a mixture of (100) b.c.c crystallized ((b) in Fig. 4c), (111) f.c.c crystallized ((c) in Fig. 4c) [21–24], and amorphous layers ((d) in Fig. 4c). In particular, this b.c.c crystallinity degradation (i.e., a mixture of b.c.c, f.c.c crystallized, and amorphous layers) would abruptly reduce the TMR ratio of the p-MTJ since it leads to a rapid decrease in Δ1 coherent tunneling of the MgO tunneling barrier [25–29]. Furthermore, for the p-MTJ spin valve with a nanoscale-thick top Co_2_Fe_6_B_2_ free layer, when the ex situ annealing temperature increased from 350 to 400 °C, the Co_2_Fe_6_B_2_ pinned layer was delineated as a b.c.c crystallized layer due to the texturing of the b.c.c bridging layer during ex situ annealing at 400 °C ((a) in Fig. 4d). The MgO tunneling barrier was also exposed as a b.c.c crystallized layer ((b) in Fig. 4d) including a local amorphous layer ((c) in Fig. 4d) and was flexuously grown. In summary, when the ex situ annealing temperature increased from 350 to 400 °C, the b.c.c crystallinity of the MgO tunneling barrier rapidly degraded for the p-MTJ spin valve with a nanoscale-thick bottom Co_2_Fe_6_B_2_ free layer but only slightly degraded for the p-MTJ spin valve with a nanoscale-thick top Co_2_Fe_6_B_2_ free layer.

**Fig. 5:**
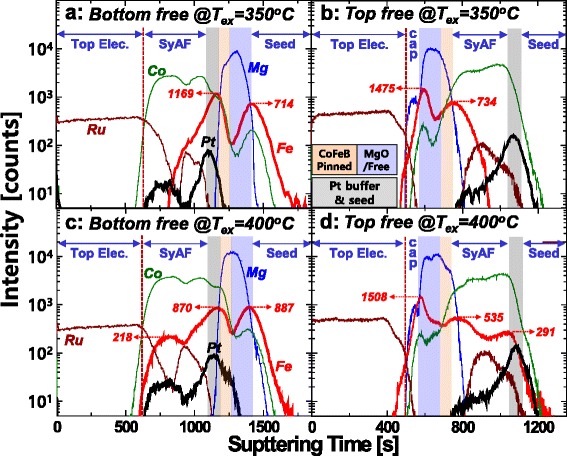
Dependency of the atomic compositional depth profiles on the p-MTJ spin valve structure and ex situ annealing temperature. **a** p-MTJ spin valve with a nanoscale-thick bottom Co_2_Fe_6_B_2_ free layer ex situ annealed at 350 °C, **b** p-MTJ spin valve with a nanoscale-thick top Co_2_Fe_6_B_2_ free layer ex situ annealed at 350 °C, **c** p-MTJ spin valve with a nanoscale-thick bottom Co_2_Fe_6_B_2_ free layer ex situ annealed at 400 °C, and **d** p-MTJ spin valve with a nanoscale-thick top Co_2_Fe_6_B_2_ free layer ex situ annealed at 400 °C

To find out why the i-PMA magnetic moment of the Co_2_Fe_6_B_2_ pinned layer and the b.c.c crystallinity of the MgO barrier degraded when the ex situ annealing temperature increased from 350 to 400 °C and why these phenomena are more severe for the p-MTJ spin valve with a nanoscale-thick bottom Co_2_Fe_6_B_2_ free layer than for that with a nanoscale-thick top Co_2_Fe_6_B_2_ free layer, an atomic compositional depth profile of p-MTJ spin valve was investigated by using secondary ion mass spectroscopy (SIMS), as shown in Fig. 5. For the p-MTJ spin valve with a nanoscale-thick bottom Co_2_Fe_6_B_2_ free layer ex situ annealed at 350 °C, the relative Fe concentration of the Co_2_Fe_6_B_2_ pinned layer (1169 counts) was higher than that of the Co_2_Fe_6_B_2_ free layer (714 counts), as shown in Fig. 5a. Note that the i-PMA magnetic moment of the Co_2_Fe_6_B_2_ pinned layer was almost the same as that of the Co_2_Fe_6_B_2_ free layer when the 0.4-nm-thick Fe layer was inserted between the Co_2_Fe_6_B_2_ pinned layer and the MgO tunneling barrier. This is because the magnetic moment of the Co_2_Fe_6_B_2_ free layer sputtered on a b.c.c seed was different from that of the Co_2_Fe_6_B_2_ pinned layer sputtered on the MgO tunneling barrier, as shown in Additional file 1: Figure S1. In addition, the Pt atoms of the Pt buffer layer were diffused closely into the MgO tunneling barrier. Otherwise, for the p-MTJ spin valve with a nanoscale-thick top Co_2_Fe_6_B_2_ free layer ex situ annealed at 350 °C, the relative Fe concentration of the Co_2_Fe_6_B_2_ free layer (1475 counts) was also higher than that of the Co_2_Fe_6_B_2_ pinned layer (734 counts) and the Pt atoms of a SyAF layer did not diffuse into the MgO tunneling barrier, as shown in Fig. 5b. Remember that a 0.4-nm-thick Fe layer was always inserted in the MgO tunneling barrier. Thus, the i-PMA magnetic moment of the Co_2_Fe_6_B_2_ pinned layer ferro-coupled with the lower SyAF layer for the p-MTJ spin valve with a nanoscale-thick bottom Co_2_Fe_6_B_2_ free layer (550 μemu) was almost the same as that for the p-MTJ spin valve with a nanoscale-thick top Co_2_Fe_6_B_2_ free layer (550 μemu). Considering both the i-PMA magnetic moment of the Co_2_Fe_6_B_2_ pinned layer and the crystallinity of the MgO tunneling barrier leads to the TMR ratio for the p-MTJ spin valve with a nanoscale-thick bottom Co_2_Fe_6_B_2_ free layer (~160 %) being very slightly higher than that for the p-MTJ spin valve with a nanoscale-thick top Co_2_Fe_6_B_2_ free layer (~158 %). Remember that the MgO tunneling barrier for the p-MTJ spin valve with a nanoscale-thick bottom Co_2_Fe_6_B_2_ free layer was flat while that for the p-MTJ spin valve with a nanoscale-thick top Co_2_Fe_6_B_2_ free layer was flexuous, as shown in Fig. 4a, b. However, for the p-MTJ spin valve with a nanoscale-thick bottom Co_2_Fe_6_B_2_ free layer, when the ex situ annealing temperature increased from 350 to 400 °C, the Fe atoms of the Co_2_Fe_6_B_2_ pinned layer diffused into the SyAF layer as well as the MgO tunneling barrier, as shown in Fig. 5c. As a result, the Fe concentration of the Co_2_Fe_6_B_2_ pinned layer was decreased from 1169 to 870 counts while that of the Co_2_Fe_6_B_2_ free layer was increased from 714 to 887 counts, thereby decreasing the i-PMA magnetic moment of the Co_2_Fe_6_B_2_ pinned layer ferro-coupled with the lower SyAF layer from 550 to 450 μemu and increasing the i-PMA magnetic moment of the Co_2_Fe_6_B_2_ free layer from 85 to 90 μemu, as shown in Fig. 3a, c. In particular, when the ex situ annealing temperature increased from 350 to 400 °C, the Pt atoms of the Pt buffer layer diffused into the MgO tunneling barrier, thereby greatly degrading the b.c.c crystallinity of the MgO tunneling barrier, as shown in Fig. 4a, c. Local f.c.c layers in the MgO tunneling barrier in Fig. 4c evidently originated from the diffusion of the Pt atoms from the Pt buffer layer. Thus, for the p-MTJ spin valve with a nanoscale-thick bottom Co_2_Fe_6_B_2_ free layer, when the ex situ annealing temperature increased from 350 to 400 °C, the abrupt TMR ratio decrease from ~160 to ~72 % was obviously associated with both the i-PMA magnetic moment decrease of the Co_2_Fe_6_B_2_ pinned layer and the b.c.c crystallinity degradation of the MgO tunneling barrier. Furthermore, for the p-MTJ spin valve with a nanoscale-thick top Co_2_Fe_6_B_2_ free layer, when the ex situ annealing temperature increased from 350 to 400 °C, the Fe atoms of the Co_2_Fe_6_B_2_ pinned layer also diffused into the SyAF layer as well as the MgO tunneling barrier, as shown in Fig. 5d. As a result, the Fe concentration of the Co_2_Fe_6_B_2_ pinned layer was decreased from 734 to 535 counts while that of the Co_2_Fe_6_B_2_ free layer was increased from 1475 to 1508 counts, thereby decreasing the i-PMA magnetic moment of the Co_2_Fe_6_B_2_ pinned layer ferro-coupled with the lower SyAF layer from 550 to 480 μemu and increasing the i-PMA magnetic moment of the Co_2_Fe_6_B_2_ free layer from 82 to 88 μemu, as shown in Fig. 3b, d. In particular, although the ex situ annealing temperature increased from 350 to 400 °C, almost no Pt atoms of a SyAF layer diffuse into the MgO tunneling barrier; thus, a slight b.c.c crystallinity degradation of the MgO tunneling barrier in Fig. 4d would be related to the Fe atom diffusion of the Co_2_Fe_6_B_2_ pinned layer rather than the Pt atom diffusion of a SyAF layer. As a result, for the p-MTJ spin valve with a nanoscale-thick top Co_2_Fe_6_B_2_ free layer, when the ex situ annealing temperature increased from 350 to 400 °C, the slight TMR ratio decrease from ~158 to ~143 % was evidently associated with both the i-PMA magnetic moment decrease of the Co_2_Fe_6_B_2_ pinned layer and the slight b.c.c crystallinity degradation of the MgO tunneling barrier. The atomic compositional depth profile comparison between Fig. 5c and d clearly indicates that the Pt atoms diffusing into the MgO tunneling barrier when the ex situ annealing temperature increased from 350 to 400 °C would be a key factor in the TMR ratio degrading rapidly for the p-MTJ spin valve with a nanoscale-thick bottom Co_2_Fe_6_B_2_ free layer (i.e., ~160 to ~72 %) since the Pt atom diffusion into the MgO tunneling barrier intensively degrades the b.c.c crystallinity of the MgO tunneling barrier. In general, it has been reported that Pt has a fast diffusivity and high solubility in metals and inorganic materials [30]. The diffusion distance of Pt atoms during an ex situ annealing depends on solvent material property and the existing strain at the solvent material and Pt layer. In Fig. 5, the diffusion distance of Pt atoms into Ta buffer layer for top Co_2_Fe_6_B_2_ free layer structure was longer than that into b.c.c bridge and Co_2_Fe_6_B_2_ pinned layer for the bottom Co_2_Fe_6_B_2_ free layer structure, which is related to solvent material difference. However, for the top Co_2_Fe_6_B_2_ free layer structure, the Pt atoms did not diffuse into the Co_2_Fe_6_B_2_ pinned layer since the Pt atoms diffused through the [Co/Pt]_n_-SyAF layer.

## Conclusions

To achieve a higher TMR ratio at the BEOL temperature of 400 °C, i-PMA characteristics (i.e., i-PMA magnetic moment) of both Co_2_Fe_6_B_2_ free and pinned layers and the b.c.c crystallinity of the MgO tunneling barrier should be prevented as much as possible from degrading. The design of the p-MTJ spin valve structure is a key to meet these goals. For the p-MTJ spin valve with a nanoscale-thick bottom Co_2_Fe_6_B_2_ free layer (Fig. 1a), when the ex situ annealing temperature increased from 350 to 400 °C, the Pt atoms diffused into the MgO tunneling barrier, so the MgO tunneling barrier was transformed from a b.c.c crystallized layer into a mixture layer with local b.c.c, f.c.c crystallized and amorphous layers, thereby abruptly decreasing the TMR ratio because of the much less *Δ*1 coherent tunneling of the MgO tunneling barrier. A solution to this issue is a design of the p-MTJ spin valve with a nanoscale-thick top Co_2_Fe_6_B_2_ free layer (Fig. 1b) since it could prevent the Pt atoms diffusing into the MgO tunneling barrier because of non-necessity of a Pt buffer layer (~2.0 nm), resulting in a maximum TMR ratio at the BEOL temperature of 400 °C. In addition, for p-MTJ spin valves, ex situ annealing at the BEOL temperature of 400 °C induces the Fe atoms to diffuse into a SyAF layer, so the i-PMA characteristic (i-PMA magnetic moment) degrades considerably, thereby slightly decreasing the TMR ratio. Thus, further study is necessary to minimize the Fe atom diffusion of the Co_2_Fe_6_B_2_ pinned layer for p-MTJ spin valves.

## Additional File

Additional file 1: Figure S1. Dependency of the magnetization (*M*_*S*_**t*) and thickness of Co_2_Fe_6_B_2_ dead layer (*t*_*DL*_) on the MgO-Co_2_Fe_6_B_2_ PMA structure with Fe inserted layer. a PMA structure with single Co_2_Fe_6_B_2_ and Co_2_Fe_6_B_2_/Fe free layer on Ta seed layer, b PMA structure with single Co2Fe6B2 and Fe/Co_2_Fe_6_B_2_ pinned layer on MgO tunneling barrier. (PDF 72.6 KB)
