# Supplementary material for: Tunneling-Magnetoresistance Ratio Comparison of MgO-Based Perpendicular-Magnetic-Tunneling-Junction Spin Valve Between Top and Bottom Co2Fe6B2 Free Layer Structure
Source: Nanoscale Res Lett. 2016 Sep 27;11:433. doi: 10.1186/s11671-016-1637-9 (PMC5039147; doi:10.1186/s11671-016-1637-9)
Supplement: Additional file 1: Figure S1. — Dependency of the magnetization (M S*t) and thickness of Co2Fe6B2 dead layer (t DL) on the MgO-Co2Fe6B2 PMA structure with Fe inserted layer. a PMA structure with single Co2Fe6B2 and Co2Fe6B2/Fe free layer on Ta seed layer, b PMA structure with single Co2Fe6B2 and Fe/Co2Fe6B2 pinned layer on MgO tunneling barrier. (PDF 72.6 KB) [file 11671_2016_1637_MOESM1_ESM.pdf]

Supporting Information for

Tunneling-Magnetoresistance Ratio Comparison of Nanoscale Thick Bottom with Top $Co_2Fe_6B_2$ Free-layer for MgO Based Perpendicular-Magnetic-Tunneling-Junction with $[Co/Pt]_n$ Synthetic Anti-ferro Magnetic Layer at Back-end-of-line Temperature of 400 ^oC

Du-Yeong Lee, Seung-Eun Lee, Tae-Hun Shim and Jea-Gun Park*

†Department of Electronics and Computer Engineering, Hanyang University, Seoul, 133–791, Republic of Korea

E-mail: parkjgL@hanyang.ac.kr

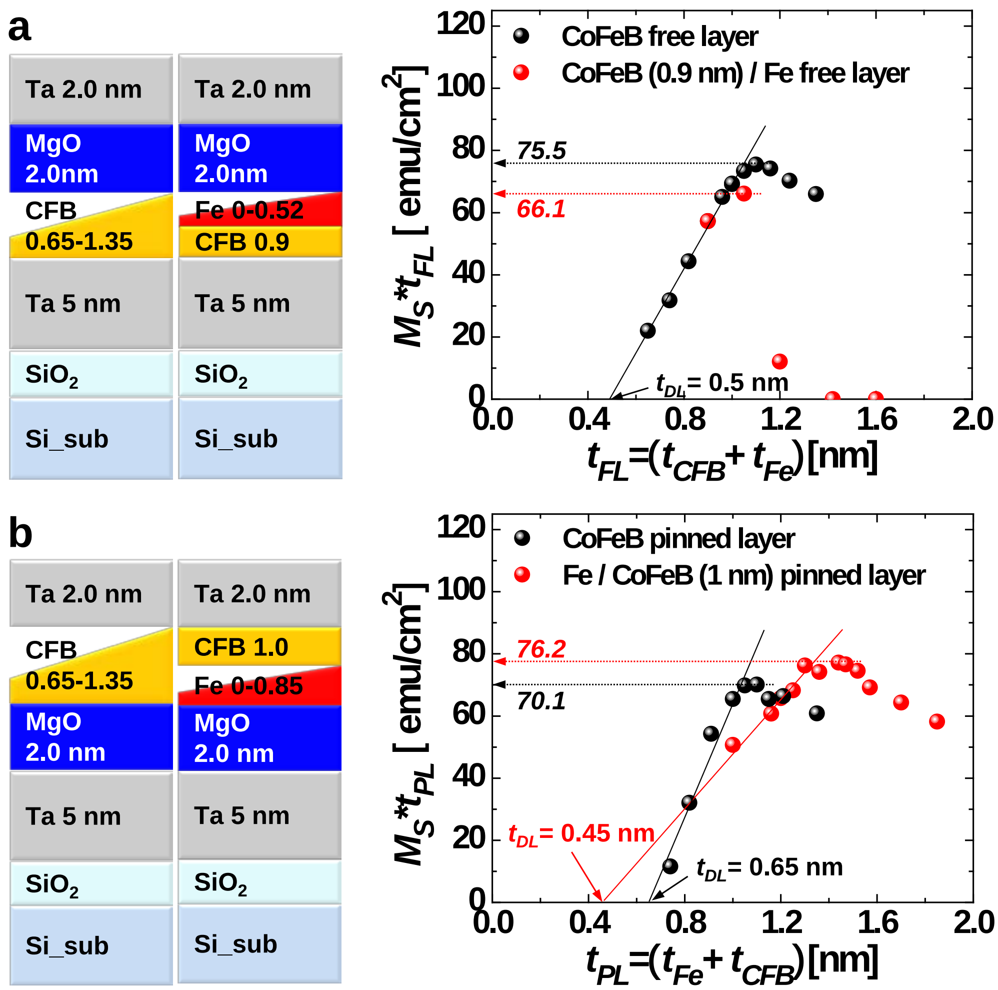

Figure Supplement 1. Dependency of the Magnetization (M_S*t) and thickness of $Co_2Fe_6B_2$ dead layer (t_{DL}) on the MgO-$Co_2Fe_6B_2$ PMA structure with Fe inserted layer. **a** PMA structure with single $Co_2Fe_6B_2$ and $Co_2Fe_6B_2$ /Fe free layer on Ta seed layer, **b** PMA structure with single $Co_2Fe_6B_2$ and Fe/$Co_2Fe_6B_2$ pinned layer on MgO tunneling barrier.
